# Completing the Pain Circuit: Recent Advances in Imaging Pain and Inflammation beyond the Central Nervous System

**DOI:** 10.5041/RMMJ.10133

**Published:** 2013-10-29

**Authors:** Clas Linnman, David Borsook

**Affiliations:** Department of Anesthesiology, Perioperative and Pain Medicine, Boston Children’s Hospital, Boston, Massachusetts, United States of America

**Keywords:** Astrocytes, inflammation, microglia, MRI, pain, PET

## Abstract

This review describes some of the recent developments in imaging aspects of pain in the periphery. It is now possible to image nerves in the cornea non-invasively, to image receptor level expression and inflammatory processes in injured tissue, to image nerves and alterations in nerve properties, to image astrocyte and glial roles in neuroinflammatory processes, and to image pain conduction functionally in the trigeminal ganglion. These advances will ultimately allow us to describe the pain pathway, from injury site to behavioral consequence, in a quantitative manner. Such a development could lead to diagnostics determining the source of pain (peripheral or central), objective monitoring of treatment progression, and, hopefully, objective biomarkers of pain.

## INTRODUCTION

Early work in the fields of neuroanatomy, neurophysiology, and clinical observations has provided a robust description of pain pathways. These pathways can now be evaluated with imaging to contribute to a more objective view of pain, where both the sensory and emotional experience may be assessed in health and disease. This review describes some recent advances in imaging of pain and inflammation-related processes below the level of the brain, that is, at the level of 1) the periphery; 2) the nerve; and 3) the nerve root. We discuss methods to measure neuroinflammation and future lines of inquiry linking peripheral markers to spine, brainstem, and brain functional imaging. An ultimate goal is a more mechanistic definition than the one currently offered by the International Association for the Study of Pain: “an unpleasant sensory and emotional experience associated with actual or potential tissue damage, or described in terms of such damage.”^1^

## IMAGING PERIPHERAL NEUROPATHY AND INFLAMMATION

Identifying active inflammatory pathology may be critical for adequate treatment. Further, precise measurement of inflammation may allow assessment of disease activity and assess the effect of therapeutic measures. Structural imaging methods such as computerized tomography (CT), magnetic resonance imaging (MRI), and ultrasound may detect large anatomical lesions and subtle swelling, but differentiating active disease from anatomical changes in healed tissue and/or normal variations is difficult. Two non-invasive imaging techniques, corneal confocal microscopy (CCM) and positron emission tomography (PET), may, however, provide insights into peripheral nerve function.

### Corneal Confocal Microscopy

The cornea is a window into free nerve fiber endings.^2^ Burning neuropathic pain and small fiber sensory loss involving the limbs, trunk, and face is characterized by abnormal skin biopsies as non-length-dependent small fiber neuropathy. A novel non-invasive technique to quantify small fiber pathology is corneal confocal microscopy (CCM). As the cornea contains C and A delta sensory fibers arising from branches of the trigeminal nerve, it offers a window for evaluating neuropathy in diabetic peripheral neuropathy,^3^ Crohn’s disease,^4^ Sjögren’s syndrome,^4^ idiopathic neuropathy,^4^ and Fabry’s disease.^5^ Future studies relating CCM findings to individual variations in pain and disability and central nervous system (CNS) function are warranted.

### Peripheral Positron Emission Tomography

Although it is not currently possible to image nociceptors *in vivo* with PET ligands directly, the technique may still inform us on the functional state of the inflammatory milieu and levels of receptor expression/occupancy. Due to changes in blood flow, vascular permeability, metabolism, white blood cell influx, and changes in the local chemical environment, many PET ligands accumulate at sites of peripheral inflammation.

**Figure 1:**
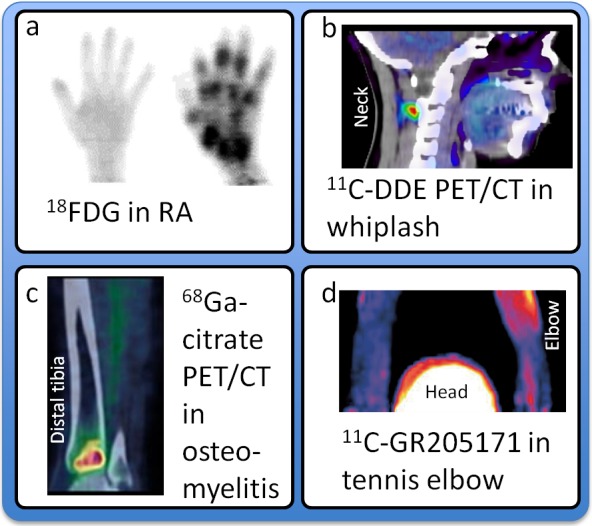
**Examples of PET Imaging of Peripheral Pain Mechanisms.** **A:**^18^F-FDG PET of the hand of a healthy subject and a patient with rheumatoid arthritis. Adapted from Beckers et al.^9^ **B:**^11^C-D-deprenyl PET/CT of a patient with whiplash-associated disorder. Adapted from Linnman et al.^11^ **C:**^68^Ga-citrate PET/CT of a patient affected by acute osteomyelitis of the left distal tibia. Adapted from Figure 6 (A ^68^Ga-citrate PET/CT scan of a patient affected by acute osteomyelitis of the left distal tibia. The scan demonstrates an area of increased tracer uptake (red area), corresponding to an area of decreased bone density on the CT images, which is consistent with acute inflammation) by Roivainen et al.^16^ with kind permission from Springer Science and Business Media. **D:**^11^C-GR205171 PET of a patient with unilateral chronic tennis elbow. Adapted from Peterson.^17^

Infection and inflammation may be visualized by scintigraphy and ^67^Gallium citrate, or autologous leukocytes labeled with indium-111 or technetium-99m.^6^ By far the most commonly used PET ligands ^18^F-fluorodeoxyglucose (FDG), thanks to its availability and its excellent properties in oncological imaging. It is a tracer for glucose metabolism, and its distribution is not specific to cancer cells but is also observed in inflammatory tissue, including macrophages, capillaries, and fibroblasts. FDG has been used to image inflammation processes and treatment monitoring in rheumatoid arthritis (Figure 1a),^7^–^9^ fever of undetermined origin (FUO), focal infection, musculoskeletal infections, sarcoidosis, and vasculitis.^10^

We have found that the tracer ^11^C-D-deprenyl provides excellent delineation of peripheral inflammatory sites, a method that holds potential to elucidate the pathophysiological mechanism in chronic musculoskeletal pain disorders, including whiplash-associated disorder (Figure 1b)^11^ and rheumatoid arthritis.^12^ The translocator protein (18 kDa) has also been targeted to image peripheral inflammation in the lung,^13^ arterial walls,^14^ and intra-plaque inflammation in carotid atherosclerosis.^15^ Other peripheral inflammation probes, such as ^68^Ga peptides targeting vascular adhesion protein 1, are being developed (Figure 1c).^16^ The use of ^68^Ga is especially interesting as the nuclide emits positrons in high yields, it is readily chelated, and it is available as a generator product rather than from a cyclotron.

The neurokinin-1 (NK1) receptor antagonist tracer ^11^C-GR205171 used for CNS imaging was recently demonstrated to show elevated unilateral uptake in chronic tennis elbow (Figure 1d).^17^ This finding suggests that NK1 receptors may be activated, or up-regulated in the peripheral, painful tissue of a chronic pain condition. The increased NK1 receptor availability is interpreted as part of ongoing neurogenic inflammation and may have correlation to the pathogenesis of chronic tennis elbow.

## IMAGING CENTRAL INFLAMMATION

Glia are the most abundant cells in the nervous system, and recent research has changed the perception of glia from being just supportive cells of neurons to being dynamic partners participating in brain metabolism and communication between neurons in health and in chronic pain.^18^–^21^

*Astrocytes* are the most abundant brain cell type in terms of their number and volume, and they constitute 40% to 50% of all glial cells. Astrocyte reaction has been demonstrated in peripheral nerve injury and in tissue inflammation models. Peripheral chronic nerve lesion is associated with breakdown of the blood–spinal cord barrier permeability and activation of astrocytes.^22^ Most animal studies have focused on the role of astrocyte activation at the spinal cord dorsal horn level, but alterations can occur at supraspinal areas, such as the rostral ventromedial medulla and in the forebrain.^23^ The enzyme monoamine oxidase (MAO)-B exists on the outer mitochondrial membrane, occurring predominantly in astrocytes.^24^ When astrocytes become activated (as customarily defined by their greatly enhanced glial fibrillary acidic protein (GFAP) binding) they express high levels of MAO-B,^25^ thereby providing an indirect target for PET imaging.

L-deprenyl (selegeline) is a selective irreversible MAO-B inhibitor that has been carbon-11-labeled, allowing for PET imaging of astrocyte activity.^26^ A deuterium substitution on the L-deprenyl molecule causes a significant reduction in the rate of trapping, thereby further enhancing the tracer’s sensitivity to subtle changes in MAO-B concentration.^27^ Thus far, studies using this deuterium-substituted deprenyl (DED) tracer have been performed to assess MAO-B function and astrocytosis in epilepsy,^28^ amyotrophic lateral sclerosis,^29^ Creutzfeldt–Jakob disease,^30^ and Alzheimer’s disease.^31^ No study to date has utilized MAO-B expression to image spinal cord or brain astrocyte involvement in human pain.

*Microglia* are the resident macrophages of the brain and spinal cord and thus act as the first and main form of active immune defense in the central nervous system. Microglia rapidly activate in response to a variety of pathological conditions, including nerve damage and persistent pain.^20^ Microglial activation is characterized by cellular responses including specific morphological changes, proliferation, increased or *de novo* expression of cell surface markers or receptors, and migration to the site of injury.^32^ Activated microglia express translocator protein (TSPO), which has been observed in animal models of neuropathic pain both in the dorsal horns of the spinal cord,^33^ the spine,^34^ and in cortex.^35^ In human studies, increased TSPO expression has been reported in the thalamus after peripheral nerve injuries^36^ and in widespread cortical regions after traumatic brain injury.^37^ PRB28, a second-generation, high-affinity TSPO radioligand suitable for imaging of microglial activation in neuroinflammation,^38^ is currently being explored for pain imaging.

MAO-B expression occurs primarily in astrocytes, while TSPO expression occurs in activated microglia and to a lesser degree in active astrocytes. Compared with the microglial response to nerve injury, astrocyte proliferation begins relatively late and progresses slowly but is sustained for more than 5 months, a time-frame paralleling the development of chronic pain.^39^ Unlike microglia, astrocytes form networks with themselves and are closely associated with neurons and blood vessels, a close contact that makes it possible for astrocytes to regulate the external chemical environment of neurons during synaptic transmission. Moreover, there is recent evidence that spinal astrocytes but not microglia contribute to the pathogenesis of painful neuropathy.^39^ Thus, the astrocyte and microglial systems are somewhat orthogonal, and site-specific PET probes may be used indicate different pathological mechanisms.

## IMAGING NERVES IN PAIN

High-resolution magnetic resonance neurography provides excellent visualization of peripheral nerves and may be an integral component in evaluating nerve injuries, supplementing electrodiagnostic (ED) studies, such as electromyography, nerve conduction studies, and quantitative neurosensory testing.^40^ Structural imaging of nerve bundles, however, has been optimized to provide 3-dimensional high-resolution and high-contrast neurography. Diffusion-weighted magnetic resonance imaging (DWI) demonstrates the random diffusion of water. By evaluating water diffusion in multiple directions, nerve fiber tracts, with their myelin sheath, may be visualized though tractography, as water diffuses along but not across the nerve bundles.^41^

**Figure 2:**
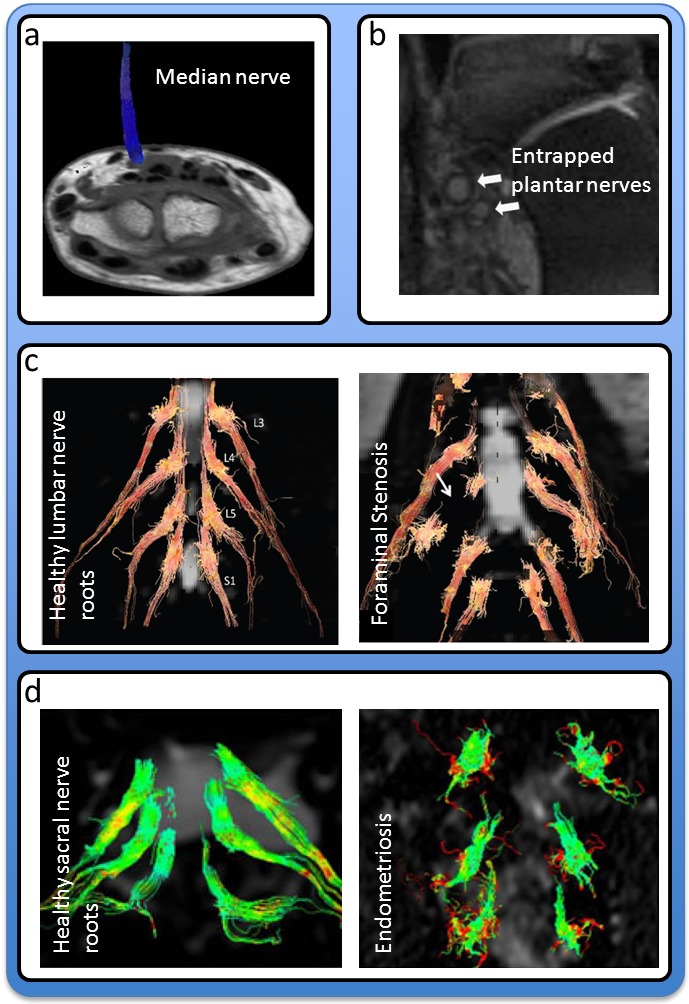
**Examples of MRI Nerve Imaging.** **a:** Tractography of the median nerve in carpal tunnel syndrome, where patients displayed a significant decrease in median nerve fractional anisotropy. Taken from Figure 2 (Tractography image demonstrating the median nerve, coded in blue, with an excellent correlation, with the reference T1-weighted image in a patient suffering from carpal tunnel syndrome) of Khalil et al.^44^ with kind permission from Springer Science and Business Media. **b:** Three-dimensional diffusion-weighted reversed fast imaging with steady-state precession (3D DWPSIF) of enlarged plantar nerves in a patient with entrapments following a repeat tarsal tunnel surgery. Taken from Figure 5b (A 32-year-old female with medial and lateral plantar nerve entrapments following a repeat tarsal tunnel surgery … Notice the depiction of the enlarged plantar nerves on the 3D DW-PSIF image) of Chhabra et al.^42^ with kind permission from Springer Science and Business Media. **c:** Lumbar nerve roots in a healthy subject and a patient with right L1-S1 foraminal stenosis, indicative of nerve root entrapment. Taken from Eguchi et al.^43^ with permission of the American Society of Neuroradiology. **d:** Fiber tracking reconstruction in a healthy woman and a patient with widespread endometriosis. Taken from Figures 2 and 3 (**2:** Example of fibre tracking reconstruction in a healthy woman showing S1, S2 and S3 nerve roots. Images are displayed in the coronal planes (radiological convention). Fibre bundles S1 to S3 display a homogeneous appearance and regular course bilaterally. **3:** Fibre tracking reconstruction in a woman affected by endometriosis of the medium and posterior compartment. The fibre bundles are short, stubby and have lots of branches.) of Manganaro et al.^45^ with kind permission from Springer Science and Business Media.

Functional imaging of the nerves and nerve roots has, to the best of our knowledge, not yet been achieved. The utility of structural and diffusion imaging of neuropathies is illustrated by a collection of prominent studies,^42^–^45^ reproduced in Figure 2.

## IMAGING NERVE ROOTS IN PAIN

**Figure 3:**
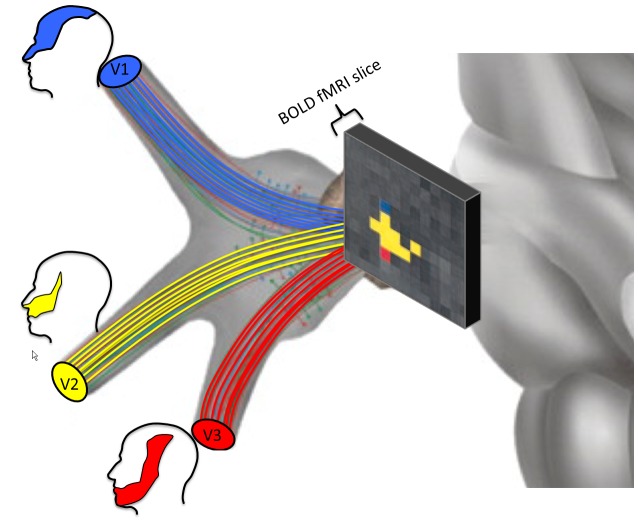
**Somatotopically Organized Activation Patterns of the Human Trigeminal Ganglion Evoked by Noxious Heat to the Ophthalmic (V1), Maxillary (V2), and Mandibular (V3) Facial Regions.** Adapted with permission from Borsook et al.^46^

Primary afferent nerves in the dorsal root ganglia convey pain information to the central nervous system. Both peripheral inflammation and nerve damage can lead to alterations in anatomy and function of neurons within the ganglion, alterations that contribute to persistent pain states.^46^,^47^ While the dorsal roots are too small for standard neuroimaging approaches, the trigeminal ganglion serves an equivalent role for the trigeminal nerve. The trigeminal ganglion is located at the base of the brain in the posterior cranial fossa across the superior border of the petrous temporal bone. It comprises sensory neurons from the ophthalmic (V1), maxillary (V2), and mandibular (V3) divisions of the trigeminal nerve. We have demonstrated that fMRI can be used to assess both sensory (brush) and noxious thermal activation of the ganglion. Activation occurred ipsilaterally and somatotopically, as predicted by the known anatomical segregation of the neurons comprising the V1, V2, and V3 divisions of the nerve (Figure 3).^46^ We have further demonstrated that sensory processing in patients with trigeminal neuropathic pain is associated with distinct activation patterns consistent with sensitization within and outside of the primary sensory pathway,^48^ and, in a case study, we demonstrated trigeminal ganglion activation in photophobia.^49^ Using diffusion tensor imaging, we have further been able to segment the peripheral trigeminal circuitry, trigeminal nerve branches (ophthalmic, maxillary, and mandibular nerves), ganglion, and nerve root, and further segment the spinal trigeminal and trigeminal thalamic tracts, which, respectively, convey information to the spinal trigeminal nuclei and ventral thalamic regions.^50^ Moreover, we have demonstrated a direct pathway from the optic nerve to the pulvinar nuclei in the posterior thalamus, providing a possible mechanism for exacerbation of pain by light in migraine.^51^ Other groups have demonstrated alterations in trigeminal nerve diffusion in trigeminal neuralgia^52^–^55^ and in temporomandibular disorder.^56^

Taken together, these studies demonstrate that, at least for cranial nerves, functional and diffusion MRI can provide mechanistic insight into pain processes at the interphase of the peripheral and central nervous system.

## SPINAL CORD PAIN IMAGING

### Positron Emission Tomography (PET)

**Figure 4:**
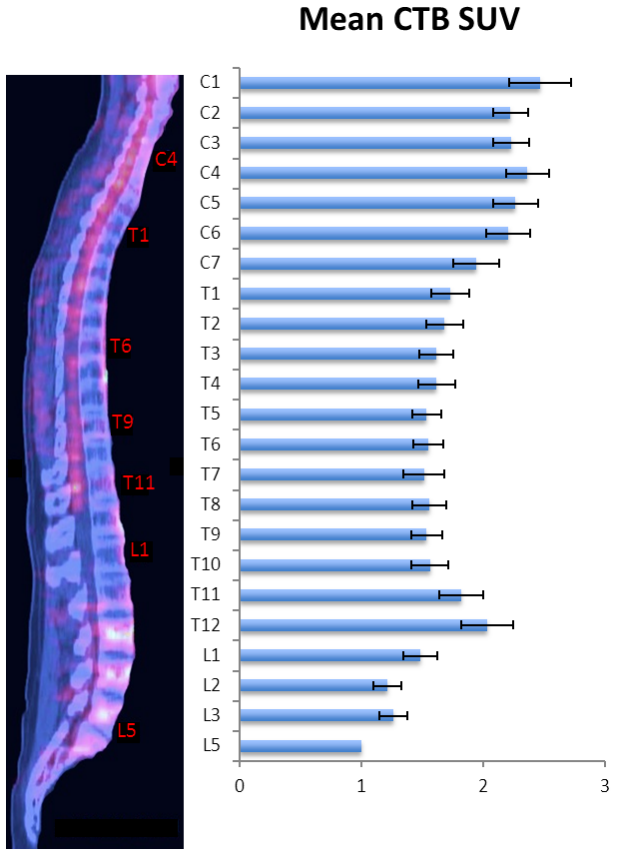
**Midline ^18^F-FDG PET/CT of a Healthy Spinal Cord.** Bar plot illustrates weighted mean cord-to-background (CTB) ratio from 122 patients.^59^,^60^

The metabolic rate of glucose increases in the spinal cord during nociceptive in-flow,^57^,^58^ affording a mechanism to image spinal pain signaling using ^18^F-fluorodeoxyglucose. We found no studies demonstrating altered spinal PET ligand uptake in pain, but such an endeavor appears possible if there is massive peripheral signaling. FDG is routinely used in oncological staging, and a retrospective analysis of cancer pain patients might demonstrate elevated FDG uptake in corresponding segments of the spinal cord. Ideally, such a study would utilize high-resolution PET in combination with MR or CT to delineate the spinal cord cross-section in multiple voxels, allowing assessment of anterior and posterior segments, and possibly lateralization effects. To illustrate PET imaging of the spine, we present mean FDG standardized uptake values (SUV) obtained from two studies of 92 patients^59^ and 30 patients^60^ without spinal malignancy (Figure 4).

### Magnetic Resonance Imaging

Structural MRI is used routinely to assess spinal cord injuries, but due to the spine’s small cross-section, and noise sources such as motion, cerebrospinal fluid (CSF) pulsation, and magnetic susceptibility, functional imaging of the spine is technically challenging. Recent developments in MR sequences and post-processing have opened up the field, and it is possible to define structure and function with greater specificity.^61^ The first functional spinal cord imaging results were published in 1999, indicating that 3-tesla imaging of the cervical spinal cord showed that repeated hand exercise led to a blood-oxygenation level dependent (BOLD)-like increase in spinal cord signal, predominantly on the ipsilateral spinal cord between C6 and T1.^62^ Since then, spinal fMRI has been reported using multiple paradigms (pain, motor, vibration, light touch) in healthy subjects and in patient populations including carpal tunnel syndrome, spinal cord injuries, and multiple sclerosis. These studies, along with methodological advances, are the subject of two excellent reviews on state-of-the-art spinal cord imaging methods^63^ and applications^64^ that we refer the reader to for full details.

## COMPLETING THE PAIN CIRCUIT: CENTRAL NETWORKS

There are only a handful of studies that have attempted to relate peripheral alterations to CNS dysfunction. An excellent example of such an approach is determining the relationship between carpal tunnel nerve conduction velocity and regional gray matter alterations in the brain.^65^ This study found that patients with carpal tunnel syndrome had significant gray matter reductions in the hand area of the somatosensory cortex, a reduction that was correlated to lower median nerve conduction velocity. Of note, diffusion tensor imaging (DTI) of the medial nerve pre and post carpal tunnel surgery indicates that postoperative clinical improvement is related to nerve diffusivity but not anisotropy.^66^ A next step may be to combine peripheral MR neurography with CNS imaging of brain morphology and function to evaluate how and when the periphery and CNS are affected by treatment. Another recent example used a combined analyte, behavioral, and imaging assessment of a rat sciatic nerve injury model to provide a “pathophysiological signature”; results indicate that the nerve injury was reflected in peripheral and central soft tissues, as well as in the expression of circulating cytokines, chemokines, and growth factors.^67^

**Figure 5:**
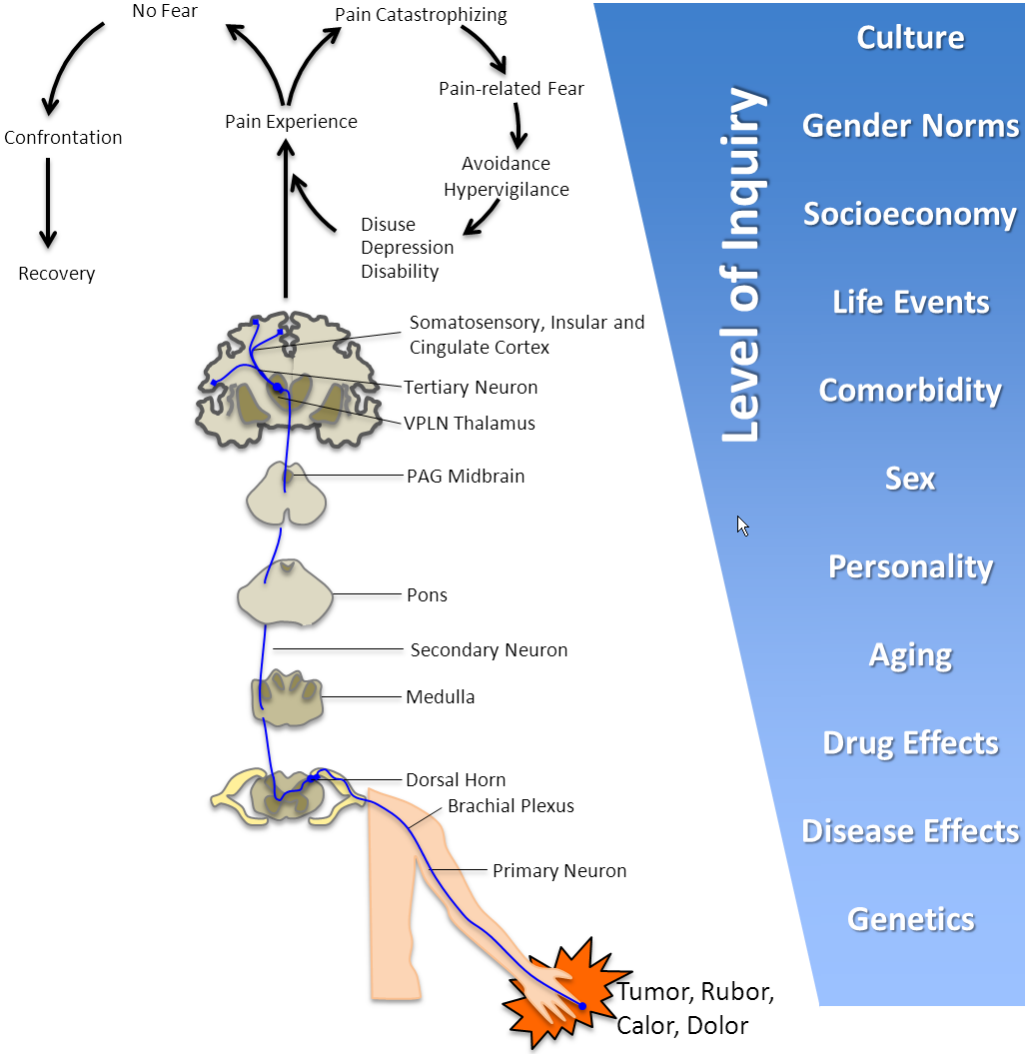
**An Illustration of the Levels of Inquiry in Pain Behavior That Imaging Has the Possibility to Inform Upon.** The Fear-Avoidance Model is an adaptation from Vlaeyen et al. and Lethem et al.^69^,^70^ Notably, inflammatory processes may interact at several levels of the pain behavior circuit. For example, the catechol-O-methyltransferase (COMT) val158met polymorphism may influence the neuronal^71^ and opioidergic^72^ response to pain (but see also Nicholl et al.^73^). Furthermore, the 18-kDa translocator protein (TSPO) Ala147Thr genotype asserts a strong influence on the binding affinity of microglial PET tracers^74^ and thus needs to be accounted for in between subject analyses. At the other end of the spectrum, imaging studies are beginning to explore how culture may interact with brain processing of perception and emotional valuation.^75^

Functional MRI and machine-learning pattern recognition can be used to define neurologic signature of acute pain with high sensitivity and specificity.^68^ The hardware (3T MRI, PET, and PET-MR), scanning sequences (structural, diffusion, BOLD, and spectroscopy), and analytical software now available have allowed the research community to quantify several aspects of the pain circuit,^69^–^75^ as illustrated in Figure 5. This circuitry is further linked to behavioral and psychological measures of pain experience, pain-related behaviors, and pain-induced co-morbidities and risk factors such as catastrophizing, fear of movement, and depression. The levels of inquiry range from genetic via neurophysiological to psychological and even sociological and anthropological domains (i.e. the perception, expression, and tolerance of pain are influenced by a variety of non-biological processes, such as disparities in work, economy, daily living, social life, gender norms, and cultural setting^76^–^78^).

Clearly, the study of pain is and will remain a multidisciplinary field. Animal imaging of brain systems, reviewed by Borsook and Becerra,^79^ offers the possibility of imaging awake animals and may serve as a “language of translation” between preclinical to clinical models. Human imaging, in turn, has strengthened and made objective the links between CNS neurophysiology and psychology of pain modulation. We foresee a similar development in the field of peripheral inflammation and spine imaging.

## FUTURE DIRECTIONS

An increasing body of literature has implicated inflammation as a contributor to the initiation but also to the maintenance of chronic pain, whether it be an obvious inflammatory process produced by specific disease (e.g. rheumatoid arthritis, chronic pancreatitis), following trauma (e.g. post-surgical neuroinflammation), or other neuropathic conditions such as complex regional pain syndrome (CRPS). It seems increasingly clear that peripheral inflammation may produce central inflammatory processes.^80^–^85^ In addition, central inflammation—even in brain areas rarely considered to be involved in chronic pain, for example in the hippocampus—produce neuropathic-like behavior in rats.^86^ In humans such changes in brain neuroinflammation contribute to altered pain^87^ and mood changes.^88^,^89^ Thus, the ability to measure neuroinflammation in humans with pain in both the peripheral and central nervous systems may provide objective indices for: 1) ongoing inflammation that may produce the maintenance of the disease either in the periphery^83^ or centrally;^36^ and 2) objective measures for treatment effects. While imaging markers may provide an initial definition of the status of inflammation, blood or serum markers may eventually be more sensitive and provide a more cost-effective use in the clinic.

## Abbreviations:

3D DWPSIF: three-dimensional diffusion-weighted reversed fast imaging with steady-state precession;
CCM: corneal confocal microscopy;
CSF: cerebrospinal fluid;
CNS: central nervous system;
CRPS: complex regional pain syndrome;
CT: computerized tomography;
DED: deuterium-substituted deprenyl;
DWI: diffusion-weighted magnetic resonance imaging;
DTI: diffusion tensor imaging;
ED: electrodiagnostic;
FDG: fluorodeoxyglucose;
FUO: fever of undetermined origin;
GFAP: glial fibrillary acidic protein;
MAO-B: monoamine oxidase B;
MRI: magnetic resonance imaging;
NK1: neurokinin-1;
PET: positron emission tomography;
SUV: standardized uptake value;
TSPO: translocator protein.
